# Treatment of Uterine Hemorrhage

**Published:** 1892-10

**Authors:** 


TREATMENT OF UTERINE HEMORRHAGE.—Routh (*Practitioner*, July 1892, p. 5) recommends that in case of profuse menorrhagia, and especially if metrorrhagia is also present without obvious cause, the cavity of the uterus should be explored. The best means of exploration is rapid dilatation of the cervix with graduated bougies, under anesthesia. With rigid antisepsis there is practically no risk and rarely consecutive pyrexia, unless malignant disease or salpingitis be present. Even if tubal disease is present or suspected, exploratory dilatation of the cervix for hemorrhage of apparently intra-uterine origin is not necessarily contra-indicated, salpingitis often being secondary to and aggravated by intra-uterine disease. If fibroids of the uterus are evidently present, the immediate cause of the hemorrhage may be a removable one, such as co-existing polypus or fungous endometritis. The uterine cavity should therefore, when practicable, be explored before removal of the appendages or hysterectomy is considered. In some cases, dilatation alone suffices to relieve hemorrhage and pain. If exploratory dilatation were more commonly adopted prior to the employment of Apostoli's treatment, it would lead to a more exact knowledge of the applicability of the latter and place its use on a more scientific basis.—*The American Practitioner and News.*

